# Mitigating Reasons for the Poor Performance of n‐CdS/p‐SnS Solar Cells

**DOI:** 10.1002/gch2.201800017

**Published:** 2018-05-24

**Authors:** Yashika Gupta, Chhaya Ravikant, Arun Palakkandy

**Affiliations:** ^1^ Material Science Research Lab S.G.T.B. Khalsa College University of Delhi, New Delhi Delhi 110007 India; ^2^ Department of Electronics Science University of Delhi‐South Campus, New Delhi Delhi 110021 India; ^3^ Department of Applied Sciences Indira Gandhi Delhi Technical University Kashmere Gate, New Delhi Delhi 110006 India

**Keywords:** conversion eficiency, n‐CdS/p‐SnS solar cells, trap‐assisted tunneling recombination

## Abstract

In the present work, indium tin oxide (ITO)/n‐CdS/p‐SnS/Au structured solar cells are fabricated with best conversion efficiency of 0.005%. A detailed investigation is made into the cause of the poor conversion efficiency and the cause is narrowed down to defects in p‐SnS which effect the junction and the neutral region of the cell. The junctions performance is quantified using the ideality factor which is found to be related to the band misalignment. The paper also investigates into literature and discusses efforts made to overcome the problems with this structure.

## 1 Introduction

Tin sulfide (SnS) promises to be one of the most suitable inorganic, nontoxic, readily available material suitable for photovoltaic application in thin film state.^1^, ^2^, ^3^ The structural, optical and electrical properties of SnS thin films such as grain size and orientation,^1^, ^4^ conductivity,^5^, ^6^ band‐gap,^7^, ^8^ etc. can be easily tuned by controlling the fabrication conditions. On top of this, Loferski et al.^9^ using theoretical simulations claim a possible conversion efficiency of 24% for SnS solar cells. The first ever solar cell using SnS was fabricated by Noguchi et al.^10^ in 1994 with a cadmium sulfide (CdS) window layer. Over the last two decades (1994–2016), conversion efficiencies ranging from 0.0025% to 0.29% have been reported for pristine CdS/SnS solar cells.^10^, ^11^, ^12^, ^13^ This is far less than the Lofereski's claim.^9^ However, recently researchers have reported efficiencies greater than 2.5% for CdS/SnS heterojunction solar cells by including an extra Zn‐based (mainly ZnO) buffer layer in the structure or inverting the cell geometry.^14^, ^15^, ^16^, ^17^ Although the p‐SnS solar cell performance has improved by adding the extra buffer layer, these results fail to comment upon the factors effecting the pristine CdS/SnS junction which leads to the need of a buffer layer.

This, hence implies serious gaps exist in our understanding of p‐SnS based heterojunction solar cells which needs to be addressed in order to improve SnS based solar cell's performance. A solar cell consists of three regions, namely a) the junction region, b) the neutral region, and finally c) the electrodes. A drop in performance of any one of the three regions would result in a substantial drop in the device performance. In our previous report,^18^ detailing our extensive study on p‐SnS thin films properties as a function of film thickness, we had shown that the diffusion length of holes through the neutral region would play an important role in deciding the solar cell's conversion efficiency. Based on the results of that study, we claimed that a p‐SnS active layer of 900 nm thickness would be best suited for solar cell application. The aim of this manuscript is to study the effect of SnS thin film properties on the heterojunction formed with CdS layer and to investigate the reasons for the poor efficiency reported to date for pristine n‐CdS/p‐SnS solar cells.

## 2 Solar Cell Fabrication

**Figure 1:**
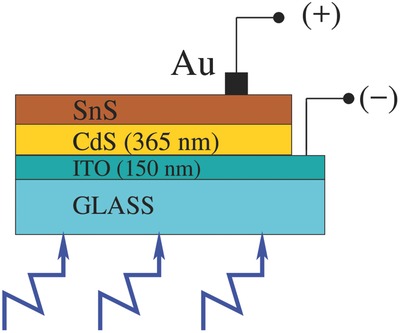
Schematics of ITO/n‐CdS/p‐SnS/Au solar cell fabricated for this study.

Thin films of CdS and SnS were fabricated in our lab on indium tin oxide (ITO) substrate by thermal evaporation using a Hind Hivac (12AUD) coating unit, in vacuum better than 10^−5^ Torr. The layers were fabricated one after another as per the schematics shown in **Figure** **1**. The window layer, CdS films grown on the etched ITO layer of 150 nm. The etching was done to prepare ITO as the front transparent electrode of the solar cell. CdS thin film grown at room temperature is invariably n‐type.^19^ On this CdS layer, p‐SnS active layer was grown. The thickness of CdS layer was controlled during evaporation using a digital quartz monitor (and subsequently verified with Dektek surface profilometry) at 365 nm in this study. CdS layer thickness was selected on the basis of literature on n‐CdS/p‐SnS heterojunction solar cells having same geometry as the present work.^12^, ^13^, ^20^ To study the behavior of n‐CdS/p‐SnS structure as a function of p‐SnS film thickness, p‐SnS layers of different thicknesses were fabricated on top of the CdS films. Since SnS films take‐up p‐type conductivity when grown on heated substrate,^12^ the ITO/n‐CdS were kept at 180 °C. The nature of film conductivity were verified by hot‐probe method. Finally, a gold electrode was grown on the p‐SnS surface using dc‐sputtering. Structural and optical studies of the films were carried out using a Bruker D8 X‐ray diffractometer (with a Cu target, λ ≈ 1.5406 Å) and Systronics double beam UV–vis spectroscopy (in the wavelength range of 300–900 nm), respectively. The *JV* characteristics were measured using a Keithley's 2400 source meter under standard AM 1.5 spectra.

## 3 Results and Discussion

### 3.1 Structural Analysis

**Figure 2:**
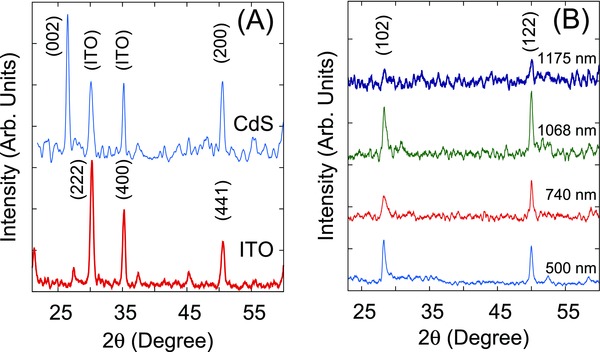
A) Representative X‐ray diffractogram of CdS film on ITO substrate along with raw ITO substrate for comparison. B) Comparison of X‐ray diffraction patterns of p‐SnS films of varying thicknesses.

The X‐ray diffractogram of CdS film (**Figure** **2**A) showed sharp diffracting peaks indicating that CdS grown at room temperature is crystalline in nature. While the underlying ITO peaks are also present in the X‐Ray diffractograms, the peak at 2θ ≈ 26.5° is unique to CdS (ASTM card 77‐2306) and corresponding to the (002) reflecting plane. CdS, hence exists with hexagonal unit cell. Considering that the (200) peak may also contain the overlapping (441) peak of ITO, the crystallite size of CdS was calculated from the (002) peak only using the Scherrer formula. The intensity of the ITO peaks from the underlying layer are comparable to that of CdS indicating a higher degree of crystallinity of ITO in comparison to that of CdS. Calculations gave CdS crystallites to be of ≈22.2 nm.

Figure 2B exhibits the XRD diffractograms of SnS films of different thicknesses. The peak positions were found to be coinciding with those listed in ASTM card 75‐0925, indicating an orthorhombic structure of the SnS unit cell with lattice parameters *a*, *b*, and *c* equal to ≈0.3980, ≈0.433, and ≈1.118 nm, respectively. The unit cell consists of tin and sulfur atoms held by covalent bonds arranging themselves in layered structure (referred to as “*a*–*b*” plane) with van der Waal forces acting between the layers along the “*c*”‐axis. The peaks at 2θ ≈ 28° and ≈50° correspond to (102) and (122) orientation of crystal planes, respectively. Both the peaks were found to be shifted from the positions mentioned in the ASTM card, peak corresponding to (102) orientation was right‐shifted while the (122) orientation peak was left‐shifted indicating compressive and tensile stress acting on the two diffracting planes. However, X‐ray diffractogram corresponding to 1175 nm showed a drastic decrease in X‐ray peak intensities. This decrease in XRD peak intensities could be due to the change in crystal orientation or grain morphology in SnS thin films expected in films of this thickness based on our observations reported earlier.^21^ SnS films showed crystallinity with crystallite sizes in the nanoregime (≈20–30 nm). Within experimental error, we may consider that there is small or negligible variation in crystallite size with film thickness. The crystallite size hence is considered to be constant for all the film samples in this study. The XRD analysis confirms the chemical composition of CdS and SnS layers.

### 3.2 Analysis of UV–Vis Spectroscopy

The UV–vis absorption spectra of CdS layer on ITO was used to determine the bandgap of the 365 nm CdS layer, used as the window layer in our ITO/n‐CdS/p‐SnS/Au solar cell structure. Standard Tauc method^22^ was used to estimate the bandgap of CdS layers. CdS layer was found to have a direct bandgap of *E*_g_ ≈ 2.42 eV which matched those reported in most of the literature.^12^, ^23^, ^24^ The UV–vis absorption spectra of p‐SnS active layers, after baseline subtraction of glass/ITO/CdS spectrum, were used to determine the bandgap. **Table** **1** reports the bandgap of p‐SnS absorber layer of different thicknesses along with those reported in our previous study.^18^ The structural defects like Sn‐vacancies responsible for the p‐type conductivity of SnS thin films^25^ also lead to spreading of band‐edges within the forbidden energy gap,^26^ known as the Urbach tail (Δ*E*). This results in a decrease in the effective bandgap of the material. Δ*E* was calculated from the exponential region of the absorption spectra appearing just before the band edge, using the equation^27^

(1) $$\alpha =\alpha_{o}\exp\left(\frac{h\nu }{\Delta E}\right)$$

where “α” is the absorption coefficient.

**Table 1:** Table gives the bandgap of p‐SnS from the present and previous studies

Cell SnS thickness [nm]	*E*_g_ [eV]	SnS film thickness [nm] (from previous study)	*E*_g_ [eV]
500	1.95	450	1.83
740	1.66	650	1.8
1068	1.65	870	1.79
1175	1.6	960	1.74

**Figure 3:**
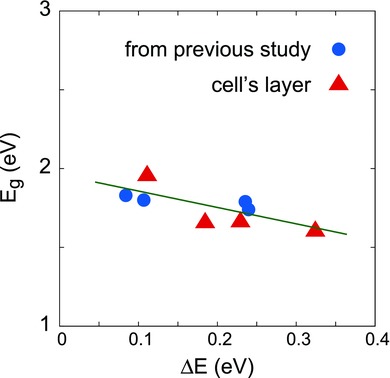
Variation of bandgap with Urbach tail. Graph contains data from both this study and that was presented in our previous work.

The bandgap of p‐SnS absorber layers was found to decrease with increasing Δ*E* as shown in **Figure** **3**. The trend obtained is similar to that obtained by Ikhmayies et al.^28^ Increased spreading of the band edges would lead to more number of localized states within the forbidden gap, which can act as trap and recombination centers for the charge carriers. In Figure 3, the triangular data points correspond to the p‐SnS active layer films of the solar cells under study. They fall on the same trend that we reported for p‐SnS thin films in our previous work (circular data points of Figure 3). This indicates good reproducibility of our results. Also, the bandgap was found to be decreasing with increasing thickness following the inverse square law relation showing the presence of quantum confinement in as‐grown films as expected. The observed increase in bandgap due to quantum confinement in SnS has also been reported earlier.^29^

### 3.3 *JV* Characteristics

**Figure 4:**
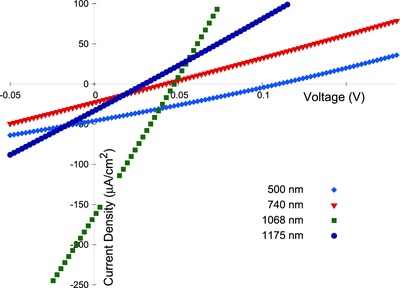
The *JV* characteristics of various solar cells with varying SnS film thickness taken exposed to light.

**Figure** **4** shows the *JV* characteristics of five different solar cells. Each ITO/n‐CdS/p‐SnS/Au structure solar cell had a different thickness of the p‐SnS active layer. The thickness of the p‐SnS layer have been indicated in the figure itself. Immediately one can make out two important features from the *JV* characteristics. The first significant feature is that the area enclosed by the *JV* curve in the fourth quadrant is not rectangular in shape but triangular. Such “near‐triangular” area under the curve in the fourth quadrant have also been observed by Li et al.^11^ and Avellanda et al.^30^ in CdS/p‐SnS solar cells. This shape is explained due to the lowering in value of the shunt resistance (*R*_sh_) or in other words increased leakage current through the solar cells junction.^31^, ^32^, ^33^, ^34^

The shunt (*R*_sh_) and series resistances (*R*_s_) for the five solar cells were calculated from the data of Figure 4 using the equations

(2) $$\begin{array}{l} R_{\mathrm{sh}}={\left(\frac{\partial V}{\partial J}\right)}_{J=J_{\mathrm{sc}}}\\ R_{s}={\left(\frac{\partial V}{\partial J}\right)}_{V=V_{\mathrm{oc}}} \end{array}$$

*R*_s_ and *R*_sh_ were found to be approximately equal and decreasing with increasing SnS layer thickness (**Figure** [qv: **5**]). This decrease in *R*_s_ with increasing film thickness could be well expected from the increasing hole mobility with film thickness of p‐SnS layer as reported in ref. 18 (i.e., resistivity decreases with film thickness) and also from the inverse relation between the resistance and area of cross section of resistive path. *R*_sh_ accounts for the leakage current in the cell. Ideally, the value of *R*_sh_ should be infinite indicating that there is no leakage current, however a lowering in *R*_sh_ value indicates the presence of a conductive path across the depletion width. This leakage path deprives current from the intended load and hence degrade the cell performance.

**Figure 5:**
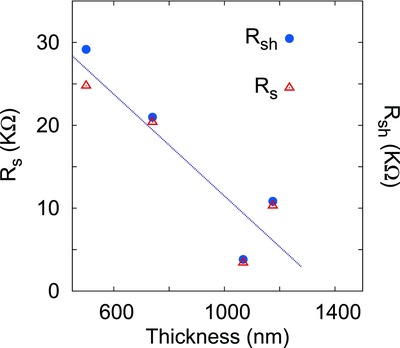
Series and shunt resistance variation with SnS layer thickness.

## 4 Diode Analysis

### 4.1 Neutral Region

**Figure 6:**
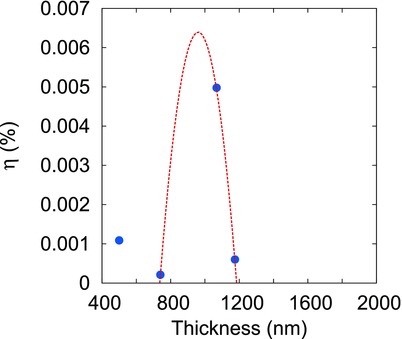
Figure showing conversion efficiency of the solar cells as a function of SnS film thickness. The trend line shown here is the quadratic fit generated using curve fitting and is made to draw a parallel with variation of diffusion length with film thickness as reported in our previous study.^18^ The maximum efficiency at ≈900 nm, similar to the diffusion length trend, confirms that the neutral region of a solar cell affects it performance.

**Figure** [qv: **6**] shows the variation in conversion efficiency with increasing absorbing layer (p‐SnS) thickness. The trend obtained for efficiency variation with film thickness corroborates our initial result of diffusion length variation of majority carriers with film thickness.^18^ The photogenerated carriers while moving toward electrodes can also face recombinations in the neutral region due to various mechanisms like Auger recombination, recombination through R‐G centers, etc. and thus become unavailable for power generation. This suggests that the neutral region's length of the cell should be optimized (considering thickness dependence of the SnS properties) such as the diffusion length of the majority carriers is maximized. In our previous study, we experimentally measured mobility (μ) and life‐time (τ) for holes in p‐SnS thin films using Hall effect and persistent photoconductivity (PPC) measurements, respectively and found that both these quantities have an opposite trend with film thickness. The life‐time, τ was found to decrease and mobility, μ to increase with film thickness, implying the existence of an optimum film thickness for which the *μτ* product or the diffusion length (*L*)^35^

(3) $$L=\sqrt{\frac{KT\mu \tau }{q}}$$

is maximized. For the p‐SnS films, the optimum thickness with maximum diffusion length was found to be ≈900 nm. The diffusion length trend was found to be decreasing for thicknesses greater or less than 900 nm. Similar to the trend seen in Figure 6. This reconfirms our initial result that along with the junction, the neutral region of a solar cell also plays an important role while deciding its conversion efficiency.

Reddy et al.^36^ claimed to have improved the CdS/SnS solar cell efficiency by reducing the resistivity of the n‐side neutral region by doping CdS layer with Indium metal, which in turn increased the mobility of the carriers. Similar approach was used by Hegde et al.^20^ and Ghosh et al.^12^ with indium and chromium doping of CdS layer, respectively to decrease the resistivity and hence improve the photovoltaic performance. Hegde et al also observed a change in bandgap of the CdS films upon Indium doping, which might have also helped in making band alignment favorable for the solar cell energy conversion. We shall see this in next section.

### 4.2 Depletion Region

One of the major reason contributing to such current leakage paths is the trap‐assisted tunneling recombination taking place in the junction.^37^, ^38^, ^39^ As already discussed, Sn vacancies give rise to shallow acceptor levels in forbidden bandgap. These levels act as trap centers and result in recombination of the photo‐generated charge carriers at the p‐SnS/CdS junction.^20^ These defects states present at the p‐SnS/CdS junction might also be the responsible for low open circuit voltages obtained for the solar cells under study as suggested in literature.^40^ Note that the point defects in CdS layer, caused by Cd‐interstitials (Cd_i_) and Cd‐antisites (Cd_s_) gives rise to shallow donor levels in the forbidden bandgap of CdS thin films.^38^ These would also contribute to trap‐assisted tunneling recombination on n‐side depletion region.^38^ However, since the CdS layer is common to all the cells, the different values of *R*_sh_ for different cells is due to the varying defect concentration for varying thicknesses of SnS layers in the cells as is evident from different band spreading (or Δ*E*) observed for the cells (Figure 3).

Apart from the recombinations due to traps, the band‐misalignment between the junction layers also results in loss of charge carriers at the junction. The offset (Δ*E*_c_ or Δ*E*_v_) in the positions of conduction band minima (CBM) or valence band maxima (VBM), respectively results in formation of a cliff or a spike structure at the junction, which is determined by solving the Poisson's equation at the junction. These structures arising due to band discontinuity at the junction impedes the carrier transport and hence deteriorates the cell performance. Figure 9 shows the energy band diagram for CdS/p‐SnS heterojunction as reported by Haleem et al.^23^ for orthorhombic SnS along with the p‐SnS/Au junction, the values of conduction band minima (CBM), valence band maxima (VBM), work function of Au metal are taken from literature.^41^, ^42^ The Δ*E*_c_ between CdS and SnS layer would lead to formation of cliff structure^43^ at the junction. Any increase in the bandgap of SnS film (due to film parameters like Δ*E*, film thickness, etc.) would lead to the shifting of CBM thus increasing the conduction band offset Δ*E*_c_ and increasing barrier/cliff height, as is clear from the figure. Also, since these structures would hinder the movement of the carriers across the junction, there would be carrier accumulation at the junction which would manifest as large ideality factor for the heterojunction. This would imply that the poor band alignment (Cliff barrier) at the SnS/CdS junction assist the defect states present at the junction and further deteriorate the solar cell performance, possibly by resulting in lower *V*_oc_ values.^40^

Hence, to study the overall effect of the junction properties (both recombination and band misalignment) on solar cell performance, we calculated ideality factor (*n*) for all the junctions under study. Since the light characteristics of the cells were dominated by the parasitic resistance effect, therefore to quantify the junction behavior “*n*” was calculated using the dark characteristics of the cell as per the model discussed in our previous work.^44^ An increasing trend was obtained between the SnS film bandgap and the ideality factor for p‐SnS/CdS heterojunction (see **Figure** [qv: **7**]) implying an increase in band‐misalignment with increasing bandgap of the SnS absorber layer. The scattering observed in the graph might be present due to different band tailing observed in the samples. To confirm our results that poor performance of CdS/SnS heterojunction is not only due to band misalignment but also due to tunnel assisted recombination taking place in the junction, we have shown the variation in efficiency with dark ideality factor taken from literature for pristine CdS/SnS junction having SnS layer thickness varying from 1 to 1.5 µm as shown in **Figure** [qv: **8**]. The exponentially decreasing efficiency of the cells with increasing ideality factor implies that the SnS defect structure not only affects the solar cells' neutral region properties but also the junction behavior.

**Figure 7:**
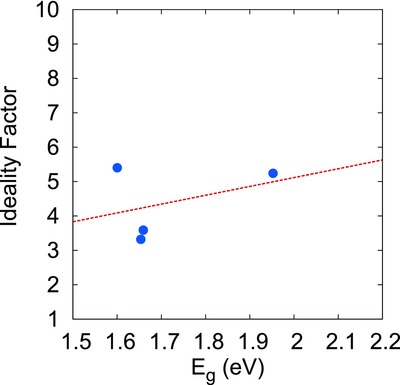
Ideality factor (*n*) variation with SnS layer bandgap *E*_g_.

**Figure 8:**
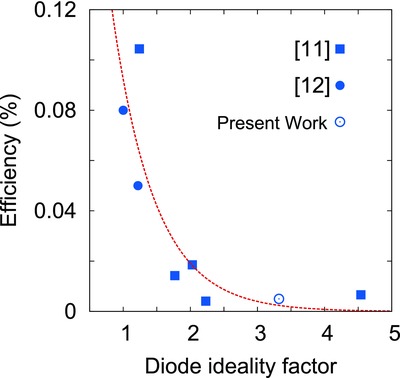
Decrease in cell efficiency with increasing ideality factor suggesting role of defects in cell performance.

The above analysis suggests that the poor performance of the p‐SnS/CdS heterojunction under‐study is due to both the defects present in the SnS film and the band‐misalignment between the two junction layers. Thus, to enhance the performance of p‐SnS based solar cells, the band‐misalignment has to be reduced for an optimized SnS layer thickness of 900 nm so as to maximize the charge carrier diffusion length and minimise the recombinations at the junction. One of the most common method used to improve band‐alignment is by doping the two layers. Since doping the SnS layers with metals results in change of its conductivity from p‐type to n‐type,^45^ doping the CdS layer is suggested for enhanced photovoltaic performance.^12^, ^20^, ^36^ Previously, Gordon et al.^43^ had worked on improving the p‐SnS solar cell's efficiency by working on the junction behavior of SnS/Zn(O,S). They optimized the S/Zn ratio of the Zn(O,S) buffer layer to 0.37 for maximum efficiency.^43^ Any increase in ratio lead to large misalignment of energy bands of the two layers while decreasing the ratio lead to triangular *J*–*V* characteristics due to high conductivity of the buffer layer in the depletion region.

We shall now look into the influence of selected electrode on the cell performance.

### 4.3 Role of Electrode

To complete the analysis and understanding of p‐SnS photovoltaic, we looked at the effect of different electrodes on the performance of our solar cells. To study the effect of electrode material, we selected our solar cells with 1068 nm thick absorber layer (p‐SnS). These cells with gold electrodes gave us the best efficiency. Instead of gold, we tested the same cells with aluminium (Al) electrodes. The *J*–*V* curve under illumination obtained for the ITO/n‐CdS/p‐SnS/Al structure showed the same “triangular” area enclosed in the forth quadrant, implying a low *R*_sh_, approximately equal to *R*_s_ as was the case with Au electrodes. As explained above, this is due to poor junction formed at the CdS–SnS interface. **Table** **2** compares the solar cell parameters for the two cells with Al and Au electrodes.

**Table 2:** Table comparing the efficiency (η), series and shunt resistance under dark conditions, ideality factor (*n*) for solar cell with 1068 nm thick SnS layer with different metal electrodes

Parameter	Cell with Au electrode	Cell with Al electrode
η [%]	0.005	0.0006
*R*_sh_ [kΩ]	62.19	49.5
*R*_s_ [kΩ]	2.0	14.6
Ideality factor	3.32	4.5

Parameters listed in Table 2 indicates the importance of electrode selection for solar performance. Both Al and Au form the ohmic junction with p‐SnS layer, however the contact resistance of Al electrode is much more than the that of Au electrode as is indicated by the increase in series resistances for the cells with Al electrodes. The increased series resistance ultimately deteriorates the solar cell efficiency by a factor of ten from 0.005% with Au to 0.0006% with Al. Another interesting observation is the increase in the ideality factor for the cell with Al electrode. Aluminium metal has a work function of 4.2 eV which is far less than the 5.3 eV work function of Au metal.^41^ As seen from the band‐diagram (see **Figure** [qv: **9**]), this would mean an increase in the barrier height at the semiconductor–metal junction for Al electrode as compared to the Au electrode. This in turn would lead to hole accumulation at the semiconductor–metal junction and hence is reflected as an increase in ideality factor. Thus, along with a good p‐SnS/buffer layer junction, p‐SnS/metal contact interface should also be optimized to minimize loss of charge carriers and recombinations^40^ for an efficient solar cell.

**Figure 9:**
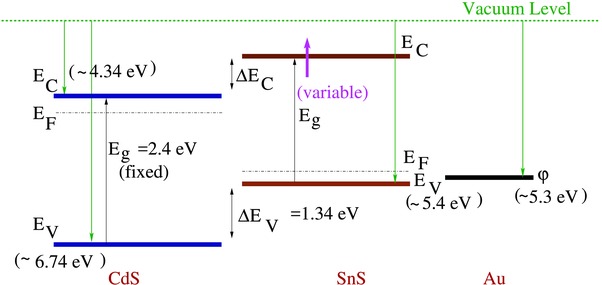
Energy band diagram of CdS/p‐SnS/Au junction.

Interestingly, Noguchi et al.^10^ achieved an efficiency of 0.29% using Silver electrode having a work function of 4.7 eV^41^ which is the highest efficiency reported for the ITO/n‐CdS/p‐SnS heterojunction solar cell structure so far. However, the results were not reproduced in any of the future studies. Ghosh et al.^12^ and Revathi et al.^46^ used silver as back electrode and got 0.08% and 0.015% efficiency, respectively which is still higher than rest of the works reported on pristine CdS/SnS solar cells. Thus, underlining the importance of proper electrode selection.

As a closing note, we would like to add that literature on CdS/SnS solar cells have suggested that a further increase in energy conversion efficiency can be obtained by annealing the cell structure at temperatures as high as 450 °C for long time^11^, ^46^ or by doping the CdS layer to increase the conductivity of the neutral region.^12^, ^20^, ^36^ However, our attempts of annealing lead to the SnS layer losing its p‐type conductivity and becoming n‐type, leading to a loss of solar cell type behavior. This p‐ to n‐type conductivity change on annealing at temperatures as low as 200–280 °C have also been reported earlier.^6^, ^47^

## 5 Conclusion

Solar cells with varying p‐SnS absorber layer thicknesses were grown in a super‐state configuration of ITO/n‐CdS/p‐SnS/Au structure. The analysis of *J*–*V* curves suggest that the defect structure of p‐SnS thin film effect both of the neutral and junction region properties of the SnS solar cells. For the given structure, maximum efficiency is obtained for the cell with absorber layer thickness of 1068 nm which is closest to the optimized thickness of 900 nm predicted by us based on the diffusion length of the majority carriers. The highest efficiency achieved in the present work is far less than the maximum reported efficiency of ≈0.3% for pristine n‐CdS/p‐SnS heterojunction. In this paper, we have evaluated the role of all the three regions of the ITO/n‐CdS/p‐SnS/Au solar cells, namely the neutral, junction and electrode regions, in cell performance. The poor performance of the cell was due to decreased shunt resistance or increased current leakage in the cell. The native defects of both the junction layers contributed in trap‐assisted tunneling recombination across the junction and hence an increase in leakage current. Also, the band‐alignment between the CdS and SnS layers is not favourable for carrier transport due to presence of large conduction band offset, impeding the flow of charge carriers toward the electrodes.

## Conflict of Interest

The authors declare no conflict of interest.
